# Trends in prediabetes and diabetes prevalence and associated risk factors in Vietnamese adults

**DOI:** 10.4178/epih.e2020029

**Published:** 2020-05-11

**Authors:** That Thanh Ton, Anh Thi Ngoc Tran, Ich Thanh Do, Hoa Nguyen, Thi Thanh Binh Nguyen, Minh Tu Nguyen, Van Anh Bao Ha, Anh Quoc Tran, Huu Khoi Hoang, Binh Thang Tran

**Affiliations:** 1 Da Nang Center for Disease Control and Prevention, Da Nang, Vietnam; 2 Department of Cancer Control and Population Health, Graduate School of Cancer Science and Policy, National Cancer Center, Goyang, Korea; 3 Hue University of Medicine and Pharmacy, Hue University, Hue, Vietnam; 4 Da Nang University of Medical Technology and Pharmacy, Da Nang, Vietnam

**Keywords:** Diabetes mellitus, Prediabetic state, Trend analysis, Risk factors, Vietnam

## Abstract

**OBJECTIVES:**

The prevalence of diabetes mellitus is rapidly increasing in Vietnam, particularly among adults aged over 45 years. This study estimated trends in diabetes and prediabetes prevalence and determined risk factors in Vietnamese adults (over 45 years).

**METHODS:**

A cross-sectional study was conducted based on data from an annual diabetes screening program among people aged 45-69 years in an urban city in central Vietnam (Da Nang). Joinpoint regression analyses were performed to calculate the annual percentage change and p_trend_-values. Multinomial logistic regression analysis was used to determine risk factors.

**RESULTS:**

In total, 3,765 men and 9,149 women were included in this analysis. The age-adjusted prevalence of diabetes and prediabetes in 2017 was 11.4% and 52.9%, respectively. The prevalence of diabetes was higher in men (15.1%) than in women (10.3%), but that of prediabetes was similar in both genders (53.4% vs. 52.8%). The prevalence of prediabetes significantly increased during the study period, whereas no upward or downward trend for diabetes was observed. The prevalence of obesity, abdominal obesity, hypertension, and dyslipidemia showed no obvious trend. Obesity, a high waist-to-hip ratio, hypertension, more severe abdominal obesity, and dyslipidemia were significantly associated with a higher risk of diabetes and prediabetes.

**CONCLUSIONS:**

Diabetes and prediabetes were more prevalent among people aged over 45 years than in the general population. Da Nang has experienced a marked increase in the prevalence of prediabetes. These findings have significant implications regarding the need for nationwide public health interventions and management aiming at diabetes prevention and control.

## INTRODUCTION

Diabetes mellitus is a chronic metabolic disease characterized by elevated blood sugar levels. It is 1 of 4 serious non-communicable diseases (NCDs) that the World Health Organization (WHO) has targeted for control [1]. Diabetes develops when an individual’s pancreas does not make enough insulin, or the body cannot effectively use the insulin it produces. Diabetes results in abnormal carbohydrate metabolism and elevated blood glucose levels, which subsequently lead to severe health complications including vision loss, leg amputation, heart attack, stroke, kidney failure, and nerve damage; furthermore, diabetes even increases the risk of developing some types of cancer [1,2]. Undiagnosed cases of diabetes or prediabetes are an emerging public health concern, as many instances are only identified after affected individuals develop serious complications [3]. Approximately 5-10% of individuals with prediabetes will develop diabetes within the next 10 years if their condition is not controlled appropriately, and they may experience diabetic complications [4]. As a result, prediabetes is a significant risk factor of diabetes, and this recognition should be combined with a thorough understanding of its natural history and interventions to control it.

Diabetes imposes a significant burden of disease in Vietnam [5]. The nationwide prevalence of diabetes was 6.0% in 2017, with approximately 5 million adults diagnosed with diabetes [1]. In the same year, diabetes accounted for 29 out of 1,000 deaths, of which 51.5% were among undiagnosed cases [6]. The economic burden of diabetes currently accounts for 12% of the gross domestic product per capita [7]. The burden of diabetes is increasing, and it deserves more attention since it constitutes one of the largest challenges facing Vietnam due to the aging population. Evidence from previous studies in Vietnam reveals that the prevalence of diabetes was higher among those aged over 40 years [8-10]. Demographic changes, coupled with income growth and a westernized lifestyle, particularly in the city of Da Nang (a coastal city that is the largest city in central Vietnam, with 1.134 million inhabitants), will change the distribution of diabetes in the future [11]. Therefore, controlling diabetes and prediabetes in high-risk people aged over 45 years old is likely to play a crucial role in the management of NCDs. However, analyses of trends in prediabetes and diabetes using primary data to suggest appropriate public health policy have not yet been conducted in Vietnam.

In the present study, we aimed to estimate the trends in prediabetes and diabetes prevalence and to determine related risk factors among Vietnamese people aged 45 years and older in Da Nang, Vietnam, from 2011 to 2017.

## MATERIALS AND METHODS

### Study population

This study employed a cross-sectional design using data extracted from the Provincial Non-Communicable Disease Screening program, specific to diabetes, over the period of 2011-2017. An annual screening program was undertaken in Da Nang, consisting of 1 sub-urban district (defined as a rural area in the study) and 6 urban districts.

In brief, the diabetes prevention program was a part of an NCDs screening program that has been implemented in some targeted provinces and cites since 2010. This program is managed by the Ministry of Health and technically supervised by the Vietnam National Hospital of Endocrinology. The funding partially comes from the budget of the national project and mostly relies on funding availability from the Provincial People’s Department. This program was carried out by the Da Nang Center for Disease Control and Prevention.

The program used a multistage sampling method: (1) 3 districts of the city of Da Nang were selected randomly based on the characteristics of different ecological regions; (2) 4-6 communes in each district were then randomly selected; (3) 10 villages were randomly selected from each commune; and (4) a list of people aged 45 and older of both genders in the studied villages was compiled from the list of total residents. From 2011 to 2017, a total of 21,318 people were randomly selected and invited, of whom 13,389 people agreed to take part in the program.

The screening program consisted of a survey about health behaviors and a health examination. We excluded non-residents of Da Nang and who did not either answer a health behavior survey questionnaire, had missing blood sample, or duplicated subjects. In total, 12,725 respondents were eligible for our present study.

### Measurements and definitions

Information was collected on the demographic characteristics of study participants, including age, gender, educational level, and residential area.

Diabetes and prediabetes measurements and classification followed the standards of the American Diabetes Association. Diabetes was defined as a fasting plasma glucose (FPG) ≥ 6.993 mmol/L or a 2-hour postload oral glucose tolerance test (OGTT) level ≥ 11.045 mmol/L. Prediabetes was classified as FPG of 5.550-6.993 mmol/L or a 2-hour postload OGTT level of 7.77-11.045 mmol/L [12]. All blood glucose tests were carried out by health professionals at commune health centers. The Johnson and Johnson SureStep OneTouch glucose meter (Johnson & Johnson, New Brunswick, NJ, USA) was used to test either the FPG level or OGTT level at the same laboratory (Da Nang Center for Disease Control and Prevention).

Hypertension was defined as a systolic blood pressure (SBP) higher than 140 mmHg or a diastolic blood pressure (DBP) exceeding 90 mmHg [13].

Body mass index (BMI) was calculated by dividing weight by height squared (kg/m^2^). Obesity was identified as a BMI ≥25 kg/m^2^, following the WHO criteria of BMI cut-off points for Asia-Pacific people [14].

Waist circumference was measured using a constant-tension tape at the umbilicus level. Hip circumference was measured at the level of the broadest border over the great trochanters. Abdominal obesity was defined as a waist circumference ≥ 80 cm for women and ≥ 94 cm for men [14,15].

Waist-to-hip ratio (WHR) was defined as the waist circumference in meters divided by the hip circumference in meters. A high WHR was classified as ≥ 0.9 for men and ≥ 0.8 for women.

Dyslipidemia was measured by the self-reported question: “Have you ever had dyslipidemia before?” and classified based on study participants’ answers (yes or no).

### Statistical analysis

The study participants’ demographic characteristics and related health status in each year were summarized using number and frequency. We calculated the crude prevalence of prediabetes and diabetes, and then employed the direct age standardization method to estimate the age-adjusted prediabetes/diabetes prevalence using the referenced age groups in the 2009 Vietnamese Census (above 45 years old), stratified by demographic characteristics. We conducted a similar analysis for prediabetes/diabetes prevalence based on FPG levels alone, details on which are provided in the Supplementary Materials 1 and 2.

Multinomial logistic regression models were performed to determine the risk factors of diabetes and prediabetes. The overall and gender-stratified models were employed separately. Binary logistic regression was also utilized for the composite outcome of interest (prediabetes and diabetes), and the results are provided in the Supplementary Material 3.

The Joinpoint software (developed by IMS, Inc. under contract for the National Cancer Institute of America) was applied to estimate the annual percentage change (APC) and p_trend_-values.

The threshold for statistical significance was set as a p-value of less than 0.05. Stata version 14 (StataCorp., College Station, TX, USA) was used for all data management and analysis.

### Ethics statement

The scientific committee at the Center for Disease Control and Prevention (formerly named: Da Nang City Preventive Medicine Center) and the Department of Health in Da Nang agreed for us to use data since 2016 (Decision No.1659/QĐ-SYT). In addition, ethical approval for the study protocol was obtained from the Ethics Committee for Biomedical Researches of Hue University of Medicine and Pharmacy, Hue, Vietnam (No. H2016/78, date of approval: May 12, 2016).

All participants were given information about screening and provided written informed consent for the annual screening program.

## RESULTS

Descriptive statistics on participation rates, socio-demographic characteristics of the study participants, and related health status during the study period are shown in Table 1. In total, 12,725 people were included in this study. The overall participation rate was 62.8%, with a significant increase from 42.8% in 2011 to 83.6% in 2017 (p_trend_ < 0.05). The highest participation rate was observed in 2015 (92.3%). Two-thirds of the sample were women (71.9%). There was a trend towards increased participation by people in the age group of 65-69 (p_trend_ < 0.05). Over 50% of the study participants had an education level of lower than high school. Most of the respondents reported living in urban areas (76.4%). The prevalence of obesity, abdominal obesity, hypertension, and dyslipidemia was stably constant (p_trend_ > 0.05).

Table 2 shows the trends in the crude and age-standardized prevalence of diabetes. The crude and adjusted prevalence of diabetes showed no significant changes (p_trend_ = 0.3 and p_trend_ = 0.2, respectively). The age-standardized prevalence for diabetes was 11.8% in 2011 and 11.4% in 2017. No significant changes in diabetes were noted according to age group, gender, residential area, or educational level (p_trend_ > 0.05).

As shown in Table 3, the unadjusted and adjusted prevalence of prediabetes significantly increased. The age-standardized prevalence of prediabetes was 29.4% in 2011 and 52.9% in 2017 (p_trend_ < 0.001), with an APC of 16.17%. Increasing trends of prediabetes were observed in all age groups, for both genders, in all urban areas, and for educational levels lower than college.

Trends in the prevalence of diabetes and prediabetes using only FPG levels are shown in Supplementary Materials 1 and 2. The age-adjusted prevalence (using FPG) was 10.4% and 53.2% for diabetes and prediabetes, respectively, in 2017. A significant increase was found in the prevalence of diabetes using the FPG definition among those aged 50-54 years and among those with an educational level of grade 9 and below. However, the prevalence of prediabetes based on FPG alone remained similar from 2011 to 2017 in men and in those with an educational attainment of college or higher.

**Figure 1.**
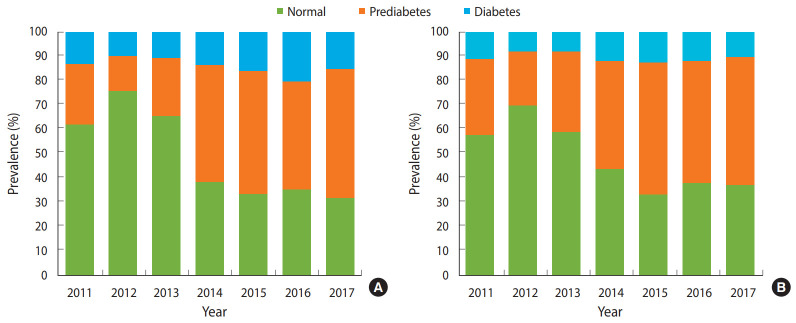
Prevalence of diabetes and prediabetes in Da Nang from 2011 to 2017, by gender (A: men, B: women).

Figure 1 shows that the disparity in the prevalence of diabetes and prediabetes by gender, which increased dramatically since 2012, when the lowest prevalence was recorded in both groups. The prevalence of prediabetes was lower in men than in women during the period 2011-2016, and then was similar in 2017 (52.8% in women and 53.4% in men). In contrast, the prevalence of diabetes remained higher in men than in women throughout the period of 2011 to 2017 (in 2017, 15.1% in men and 10.3% in women).

Table 4 shows the associations of risk factors with prediabetes and diabetes. Overall, the risk of prediabetes was higher in people with obesity (BMI ≥ 25 kg/m^2^; adjusted odds ratio [aOR], 1.53; 95% CI, 1.37 to 1.72) and hypertension (aOR, 1.10; 95% CI, 1.03 to 1.17). In contrast, men had a lower risk of prediabetes than women (aOR, 0.71; 95% CI, 0.63 to 0.80). The risk of diabetes was elevated in men (aOR, 1.22; 95% CI, 1.04 to 1.42) and individuals with obesity (aOR, 1.36; 95% CI, 1.16 to 1.58), hypertension (aOR, 1.36; 95%CI, 1.25 to 1.48), abdominal obesity (aOR, 1.53; 95% CI, 1.31 to 1.80), a high WHR (aOR, 1.65; 95% CI, 1.33 to 2.05), and dyslipidemia (aOR, 1.45; 95% CI, 1.21 to 1.73). Older age increased the risk of prediabetes and diabetes, with statistical significance in women, but no statistical significance in men. Supplementary Material 3 shows a binary logistic regression analysis of factors related to prediabetes and diabetes. The major risk factors were found to be similar to those found in the multinomial logistic regression model.

## DISCUSSION

This paper presents information on trends in the age-standardized prevalence of prediabetes and diabetes in the Vietnamese population aged 45 years and older during a 7-year period using the latest provincial screening database. Our findings revealed that about 11.4% of people had diabetes, and 1 out of 2 had prediabetes in 2017. The results of this study also indicated that the prevalence of prediabetes significantly increased in the analyzed population from 2011 to 2017, with an APC of 16.17%, whereas no significant trend in diabetes prevalence was observed in this period. Our study identified positive associations of gender, age over 60 years, obesity, a high WHR, hypertension, dyslipidemia and abdominal obesity with diabetes prevalence. Obesity, hypertension, also showed positive relationships with prediabetes.

The prevalence found in this study are higher than other recent regional estimates in the northern and southern areas of Vietnam [8,16-18] and even the national prevalence in a previous systematic review [19]. For example, a study analyzing diabetes prevalence during 2011-2013 in mountainous provinces in northern Vietnam found that 5.6% of adults aged 30-69 years old were living with diabetes [8]. Additionally, 3.7% of those aged over 40 years were found to have diabetes in a study of inhabitants of rural areas in the southwest of the Red River Delta in 2011 [20]. Our findings are consistent with those of other previous studies that also showed a higher prevalence of diabetes in Vietnam than in other Asian countries [8]. Discrepancies among studies may be at least partially due to differences in research sites, as most previous studies were conducted in rural areas. Differences in the study population are also a relevant factor, as our study only included adults aged over 45 years, which is a high-risk group. Furthermore, over the 7-year period of the study, the proportion of the 65-69 age group increased significantly, which partially contributed to the increase in the prevalence of diabetes and prediabetes. Nonetheless, our findings are similar to those of a study in Chi Linh, a northern city in which 15.9% of those aged 50-69 years were living with diabetes [10], a study in the south central coast of Vietnam that reported prevalence rates of 8.1% for diabetes and 50.1% for prediabetes [9], and a study conducted in the South Central Coast province, namely Khanh Hoa that reported a 20% prevalence of diabetes among those aged over 40 years [21]. Our results also align with those of other Asian middle-aged populations, for example, Thailand (> 13.5% with diabetes in 2014) or China (> 11.3% with diabetes in 2016) [22], Japanese adults aged over 40 years (men: 19.4%; women: 9.1%) [23], and adults in the United States [24]. Nevertheless, the estimate was lower than that of a study conducted during 2006-2014 in Hong Kong, China, which found that roughly 40% of the age group 40-79 years had diabetes [25].

Interestingly, the prevalence of diabetes did not change significantly over the 7-year period of the study (p_trend_ > 0.05), and remained stable from 11.8% in 2011 to 11.4% in 2017. This pattern is unlike the significant increasing trend found in Thailand from 2004 to 2014 [26], in Japan from 1988 to 2012 [23], and in China from 2006 to 2014 [25]. These differences may be partly due to variation in healthcare systems and services across countries. Another explanation is that our study analyzed the most recent data and covered a longer duration of follow-up than other prior studies. Our findings are contrary to those of an earlier meta-analysis showing an increasing trend in the national diabetes prevalence in Vietnam, from 2.7% in 2002 to 5.4% in 2012 [19]. This is partly a result of the diabetes prevention and management program provided in Da Nang since 2010 by the Center for Disease Control and Prevention with financial support from the Da Nang Ministry of Health. The routine annual implementation of NCDs screening programs has facilitated the early detection of prediabetes and diabetes among the population of Da Nang. Indeed, the plateauing trend of diabetes prevalence aligns with the stable trends observed in obesity and hypertension in the study population, which also agrees with a study in Thailand [26]. Our study revealed that the prevalence of diabetes based on FPG levels was lower than that based on a combination of FPG levels with OGTT results. In contrast, prediabetes appeared at a higher level. Because OGTT results were not included in the estimations, some individuals with high 2-hour postload OGTT levels may have been missed among those with prediabetes, and fewer people were diagnosed with diabetes. The finding of a significant increase in diabetes prevalence (defined using FPG levels) among adults aged 50-54 years and those with a low educational level should be highlighted.

Despite the effectiveness of diabetes control, it is remarkable that the age-standardized prevalence of prediabetes among the inhabitants of Da Nang still showed a significant increasing trend in all age groups, both genders, all educational levels, and all urban areas, and remained at a consistently high level. This trend is similar to that reported by other studies in Japan [23] and Hong Kong [25], and it is also consistent with the worldwide trend for prediabetes to become more common over the past few decades [27,28]. Urbanization in Da Nang may have contributed to an increasing threat posed by multiple lifestyle risk factors, including a sedentary lifestyle, less physical activity, eating fast food, smoking, and alcohol drinking in the most recent decade. This pattern was predominant in the 50-54 age group, which is exposed to many risk factors of prediabetes, increasing the risk that they will suffer from diabetes in their next stage of life. This finding also suggests that health education encouraging individuals to change their risk behaviors should be enhanced as a way to reduce the prediabetes rate among Da Nang residents.

The major causes of diabetes and prediabetes listed in our study were obesity (BMI ≥ 25 kg/m^2^) and hypertension in both men and women. These risk exposures have also been identified as important contributors in previous studies [5,17,29]. The odds ratio of diabetes was higher in those with a higher WHR in both men and women and in women with abdominal obesity and dyslipidemia, which is consistent with an earlier study in Vietnam [8]. Diabetes was found to be less common among those with higher educational levels, as they might have better knowledge about health information and diabetes prevention methods, spurring them to engage in healthier behaviors [26]. Diabetes was more common in men than in women; however, the converse trend was found for prediabetes, in contrast to some previous studies [8,26]. This can be explained by the fact that most of our study participants were women, who are more likely than men to have healthier behaviors and to care about their health. We also found a positive association of age with prediabetes and diabetes risk. These results are in line with those of earlier studies in Vietnam, Asia, and worldwide [24,29,30]. Our study did not identify a relationship between living areas with high glucose tolerance, as was demonstrated in previous research [19]. This may have been due to the imbalance of urban and rural populations in our study, as most of the participants lived in an urban area.

To our knowledge, this is the most up-to-date study on trends in diabetes and prediabetes prevalence among the Vietnamese population. Data were obtained through a community screening program, which was implemented consistently and comprehensively for the study population. The study was only conducted in the city of Da Nang, limiting its generalizability to the broader population. However, the feasibility of the program is currently challenged by financial constraints; thus, there are currently only a few provinces and cities in Vietnam that are regularly conducting community-based diabetes screening on an annual basis. Therefore, we believe that the results of our study can provide some good practical evidence in terms of the NCDs prevention programs.

Despite the strengths of this study, some limitations should be considered and, ideally, eliminated in further studies. Firstly, there were some limitations in our sample selection process and measurements due to a lack of financial resources. The overall response rate of 62.8% in our study was lower than the recommended rate [31], particularly in the first years of screening implementation. Additionally, the participation of women and those living in urban areas was disproportionately high in our study, with the proportion of women consistently exceeding 70% and a roughly even balance of urban and rural inhabitants only being achieved in the last 2 years of the study. These imbalances may have increased the risk of selection bias, as those who were aware of their diabetes risk were more likely to come for screening than others. This factor may have resulted in an overestimation of prediabetes prevalence. Also, due to financial constraints, only approximately half of the participants with higher risks of diabetes received 2-hour OGTT testing. Secondly, the lack of quality control in the laboratory is an important limitation of our study, with possible impacts on study results. Thirdly, we did not collect data on economic characteristics or other health behaviors, such as smoking, alcohol drinking, or diet, all of which are possible confounders. Finally, despite these promising results, our present study derives from cross-sectional observations, so it is not possible to infer causality. Further studies on this issue are therefore recommended.

Overall, this study showed higher diabetes and prediabetes prevalence in the city of Da Nang than in other provinces in Vietnam and in other Asian countries. The prevalence of prediabetes significantly increased over the 7-year study period, but that of diabetes was unchanged. We also estimated several risk factors that might have contributed to the growth in diabetes and prediabetes prevalence over the period. Despite this limitation, these findings have significant implications regarding the need for the government and professional bodies to focus on public health interventions targeting middle-aged Vietnamese adults with the aim of preventing and controlling prediabetes and diabetes nationwide.

## Figures and Tables

**Table 1.** Characteristics and health status of study participants, from 2011 to 2017 (n=12,725)

Characteristics		Total	2011	2012	2013	2014	2015	2016	2017	p_trend_
No. of invited participants (n)		21,318	4,718	5,000	6,000	1,500	1,400	1,500	1,200	
No. of examined participants (n)		13,389 (62.8)	2,019 (42.8)	3,049 (61.0)	4,026 (67.1)	1,000 (66.7)	1,292 (92.3)	999 (66.7)	1,004 (83.6)	<0.001
No. of eligible participants (n)		12,725 (95.1)	1,530 (75.8)	2,998 (98.3)	3,982 (98.9)	994 (99.4)	1,251 (96.8)	981 (98.2)	989 (98.5)	0.8
Gender										
	Women	9,149 (71.9)	1,121 (73.3)	2,159 (72.0)	2,798 (70.3)	696 (70.0)	921 (73.6)	722 (73.6)	732 (74.0)	0.4
	Men	3,576 (28.1)	409 (26.7)	839 (28.0)	1,184 (29.7)	298 (30.0)	330 (26.4)	259 (26.4)	257 (26.0)	0.4
Age (yr)										
	45–49	3,274 (25.7)	283 (18.5)	806 (26.9)	1,051 (26.4)	236 (23.7)	322 (25.7)	271 (27.6)	305 (30.8)	0.1
	50–54	2,312 (19.2)	330 (21.6)	553 (18.4)	645 (16.2)	182 (18.3)	249 (19.9)	160 (16.3)	193 (19.5)	0.6
	55–59	2,543 (20.0)	301 (19.7)	607 (20.2)	754 (18.9)	220 (22.1)	277 (22.1)	178 (18.1)	206 (20.8)	0.8
	60–64	1,979 (15.6)	246 (16.1)	477 (15.9)	564 (14.2)	160 (16.1)	194 (15.5)	183 (18.7)	155 (15.7)	0.7
	65–69	2,617 (20.5)	370 (24.2)	555 (18.5)	968 (24.3)	196 (19.7)	209 (16.7)	189 (19.3)	130 (13.1)	<0.001
Educational level										
	Illiteracy to secondary school (grade 9 and below)	7,884 (62.0)	994 (61.7)	2,008 (66.9)	2,465 (61.9)	508 (51.1)	777 (62.1)	617 (62.9)	565 (57.1)	0.5
	High school (grade 10-12)	3,277 (25.8)	442 (27.6)	661 (22.1)	1,031 (25.9)	307 (30.9)	325 (26.0)	251 (25.6)	280 (28.3)	0.5
	College or more	1,564 (12.3)	164 (10.7)	329 (11.0)	486 (12.2)	179 (18.0)	149 (11.9)	113 (11.5)	144 (14.6)	0.4
Region										
	Rural	2,999 (23.6)	0 (0.0)	1,031 (34.4)	976 (24.5)	0 (0.0)	0 (0.0)	494 (40.4)	498 (40.3)	-
	Urban	9,726 (76.4)	1,530 (100)	1,967 (65.6)	3,006 (75.5)	994 (100)	1,251 (100)	487 (49.6)	491 (49.7)	0.2
Abdominal obesity		5,807 (45.6)	737 (48.2)	1,397 (46.6)	1,927 (48.4)	395 (39.7)	636 (50.8)	390 (39.8)	325 (32.9)	0.2
Obesity (body mass index ≥25 kg/m^2^)		3,021 (23.7)	355 (23.2)	624 (20.8)	903 (22.7)	267 (26.9)	341 (27.3)	289 (29.5)	242 (24.5)	0.1
Hypertension		3,892 (30.6)	523 (34.2)	949 (31.7)	1,107 (27.8)	321 (32.3)	434 (34.7)	320 (32.6)	238 (24.1)	0.3
High waist-to-hip ratio		10,295 (80.9)	1,281 (83.7)	2,425 (80.9)	3,268 (82.1)	781 (78.6)	1,047 (83.7)	742 (75.6)	751 (75.9)	0.1
Dyslipidemia		1,549 (12.2)	149 (9.7)	493 (16.4)	417 (10.5)	144 (14.5)	139 (11.1)	101 (10.3)	106 (10.7)	0.6

Values are presented as number (%).

**Table 2.** Age-standardized prevalence of diabetes among study participants (n=12,725)^1^

Characteristics		2011	2012	2013	2014	2015	2016	2017	APC (%)	p_trend_
No. of respondents (n)		1,530	2,998	3,982	994	1,251	981	989	-	-
No. of participants with diabetes (n)		217	281	424	136	190	157	117	-	-
Prevalence										
	Crude	14.2±0.9	9.4±0.5	10.6±0.5	13.7±1.1	15.2±1.0	16.0±1.2	11.8±1.0	4.55	0.3
	Adjusted	11.8±0.9	8.6±0.5	8.8±0.5	12.1±1.1	13.5±1.0	14.2±1.1	11.4±1.0	6.23	0.2
Age (yr)										
	45–49	8.1±1.6	5.6±0.8	5.8±0.7	8.1±1.8	7.8±1.5	10.0±1.8	7.2±1.5	4.76	0.2
	50–54	11.5±1.8	8.1±1.2	7.0±1.0	9.9±2.2	12.4±2.1	11.9±2.6	12.4±2.4	10.31	0.1
	55–59	10.3±1.8	9.6±1.2	9.7±1.1	17.3±2.3	17.3±2.3	16.3±2.8	10.2±2.1	6.05	0.2
	60–64	16.3±2.4	13.2±1.6	11.7±1.4	14.4±2.8	14.9±2.6	22.4±3.1	13.5±2.8	5.50	0.3
	65–69	23.0±2.2	12.6±1.4	18.5±1.2	19.4±2.8	27.3±3.1	21.7±3.0	22.3±3.7	9.90	0.2
Gender										
	Women	11.4±1.0	8.2±0.6	8.1±0.5	11.9±1.2	12.8±1.1	12.2±1.2	10.3±1.1	4.95	0.3
	Men	13.1±1.8	9.8±1.1	10.6±1.0	13.5±2.2	15.9±2.1	20.3±2.7	15.1±2.4	4.64	0.4
Educational level										
	Illiteracy to secondary school (grade 9 and below)	12.4±1.2	8.8±0.7	10.2±0.6	12.2±1.5	13.7±1.2	15.0±1.5	11.5±1.4	5.78	0.2
	High school (grade 10-12)	9.8±1.5	8.2±1.1	7.0±0.8	12.0±1.8	14.1±1.9	14.8±2.3	12.4±1.9	10.78	0.1
	College or more	9.1±2.3	7.3±1.4	6.3±1.1	11.6±2.4	11.5±2.7	8.6±2.6	8.5±2.8	5.37	0.4
Region										
	Rural	NA	7.4±0.8	9.0±0.9	NA	NA	14.8±1.7	13.4±1.5	NA	NA
	Urban	11.8±0.9	9.2±0.7	8.7±0.5	12.1±1.1	13.5±1.0	14.7±1.7	10.3±1.0	4.87	0.3

Values are presented as %±standard error. APC, annual percent change; NA, not applicable.

1 Data are direct age adjustment of the data was done for the Vietnamese population aged ≥45 years in the year 2009.

**Table 3.** Age-standardized prevalence of prediabetes among study participants from 2011 to 2017 (n=12,725)^1^

Characteristics		2011	2012	2013	2014	2015	2016	2017	APC (%)	p_trend_
No. of respondents (n)		1,530	2,998	3,982	994	1,251	981	989	-	-
No. of participants with prediabetes (n)		469	632	1,248	540	659	489	522	-	-
Prevalence overall										
	Crude	30.7±1.2	21.1±0.7	31.3±0.7	54.3±1.6	52.7±1.4	49.8±1.6	52.8±1.6	15.24	<0.001
	Adjusted	29.4±1.3	20.2±0.8	30.6±0.8	53.4±1.7	53.3±1.5	48.7±1.7	52.9±1.6	16.17	<0.001
Age (yr)										
	45–49	25.4±2.6	19.4±1.4	29.5±1.4	49.2±3.3	52.2±2.8	46.1±3.0	47.5±2.9	14.54	<0.001
	50–54	29.1±2.5	18.1±1.6	30.2±1.8	54.4±3.7	56.6±3.1	46.9±4.0	56.5±3.6	21.24	<0.001
	55–59	32.6±2.7	19.4±1.6	29.3±1.7	56.8±3.3	52.7±3.0	50.6±3.8	57.8±3.5	16.64	<0.001
	60–64	33.7±3.0	24.3±2.0	33.0±2.0	53.8±4.0	56.2±3.6	53.6±3.7	53.5±4.0	17.29	<0.001
	65–69	32.4±2.4	25.9±1.9	34.7±1.5	58.2±3.5	45.5±3.5	53.4±3.6	50.8±4.4	16.28	<0.001
Gender										
	Women	31.1±1.5	22.3±0.9	33.3±1.0	55.6±2.0	54.2±1.7	50.3±2.0	52.8±1.9	14.09	<0.001
	Men	24.8±2.3	14.5±1.3	23.9±1.4	48.1±3.3	51.0±3.0	44.5±3.4	53.4±3.3	12.75	<0.001
Educational level										
	Illiteracy to secondary school (grade 9 and below)	31.4±1.8	20.6±1.0	31.3±1.1	53.8±2.5	55.5±1.9	49.8±2.2	55.3±2.2	16.68	<0.001
	High school (grade 10-12)	27.7±2.2	19.3±1.5	30.4±1.5	52.6±2.9	49.8±2.8	46.5±3.3	48.7±3.0	14.65	<0.001
	College or more	26.8±3.2	21.5±2.2	28.0±2.1	56.7±3.8	51.0±4.3	49.4±4.8	50.9±4.7	16.57	0.1
Region										
	Rural	NA	18.2±1.2	34.0±1.6	NA	NA	47.8±2.4	57.6±2.3	NA	NA
	Urban	29.3±1.3	21.4±1.0	29.2±0.9	53.3±1.7	53.3±1.5	49.7±2.5	48.4±2.3	16.37	<0.001

Values are presented as %±standard error. APC, annual percent change; NA. not applicable.

1 Direct age adjustment of the data was done for the Vietnamese population aged ≥45 years in the year 2009.

**Table 4.** Multinomial logistic regression analysis of factors related to diabetes and prediabetes, 2011-2017^1^

Characteristics		Total		Women		Men	
		Prediabetes	Diabetes	Prediabetes	Diabetes	Prediabetes	Diabetes
Age (yr)							
	45–49	1.00 (reference)	1.00 (reference)	1.00 (reference)	1.00 (reference)	1.00 (reference)	1.00 (reference)
	50–54	1.04 (0.89, 1.20)	1.26 (1.00, 1.60)	1.07 (0.90, 1.27)	1.29 (0.95, 1.74)	0.95 (0.71, 1.25)	1.24 (0.86, 1.79)
	55–59	1.08 (0.94, 1.25)	1.36 (1.09, 1.69)	1.07 (0.91, 1.27)	1.70 (1.29, 2.25)	1.13 (0.87, 1.47)	0.90 (0.62, 1.30)
	60–64	1.29 (1.11, 1.50)	1.70 (1.36, 2.13)	1.48 (1.24, 1.78)	2.23 (1.67, 2.98)	0.99 (0.75, 1.30)	1.05 (0.73, 1.52)
	65–69	1.30 (1.13, 1.50)	2.18 (1.77, 2.69)	1.36 (1.14, 1.61)	2.81 (2.15, 3.66)	1.21 (0.94, 1.57)	1.36 (0.97, 1.91)
Gender							
	Women	1.00 (reference)	1.00 (reference)	-	-	-	-
	Men	0.71 (0.63, 0.80)	1.22 (1.04, 1.42)	-	-	-	-
Educational level							
	Illiteracy to secondary school (grade 9 and below)	1.00 (reference)	1.00 (reference)	1.00 (reference)	1.00 (reference)	1.00 (reference)	1.00 (reference)
	High school (grade 10-12)	1.02 (0.91, 1.14)	0.85 (0.72, 1.00)	1.12 (0.98, 1.29)	0.78 (0.63, 0.97)	0.84 (0.70, 1.02)	0.96 (0.75, 1.23)
	College or more	1.12 (0.96, 1.30)	0.73 (0.58, 0.92)	1.14 (0.94, 1.39)	0.66 (0.47, 0.93)	1.05 (0.83, 1.33)	0.84 (0.60, 1.17)
Region							
	Rural	1.00 (reference)	1.00 (reference)	1.00 (reference)	1.00 (reference)	1.00 (reference)	1.00 (reference)
	Urban	1.07 (0.96, 1.20)	1.14 (0.98, 1.33)	1.07 (0.94, 1.22)	1.22 (1.00, 1.48)	1.11 (0.91, 1.35)	0.99 (0.77, 1.29)
Obesity (body mass index: ≥25 kg/m^2^)							
	No	1.00 (reference)	1.00 (reference)	1.00 (reference)	1.00 (reference)	1.00 (reference)	1.00 (reference)
	Yes	1.53 (1.37, 1.72)	1.36 (1.16, 1.58)	1.43 (1.24, 1.63)	1.33 (1.10, 1.60)	1.90 (1.51, 2.39)	1.45 (1.08, 1.95)
Waist-hip ratio							
	Normal	1.00 (reference)	1.00 (reference)	1.00 (reference)	1.00 (reference)	1.00 (reference)	1.00 (reference)
	Large	1.11 (0.97, 1.27)	1.65 (1.33, 2.05)	0.96 (0.77, 1.19)	2.38 (1.46, 3.90)	1.10 (0.98, 1.23)	1.67 (1.29, 2.15)
Hypertension							
	No	1.00 (reference)	1.00 (reference)	1.00 (reference)	1.00 (reference)	1.00 (reference)	1.00 (reference)
	Yes	1.10 (1.03, 1.17)	1.36 (1.25, 1.48)	1.09 (1.01, 1.18)	1.38 (1.24, 1.53)	1.24 (1.04, 1.50)	1.31 (1.14, 1.51)
Abdominal obesity							
	No	1.00 (reference)	1.00 (reference)	1.00 (reference)	1.00 (reference)	1.00 (reference)	1.00 (reference)
	Yes	1.07 (0.95, 1.19)	1.53 (1.31, 1.80)	1.15 (1.00, 1.31)	1.69 (1.39, 2.05)	0.85 (0.67, 1.07)	1.14 (0.85, 1.53)
Dyslipidemia							
	No	1.00 (reference)	1.00 (reference)	1.00 (reference)	1.00 (reference)	1.00 (reference)	1.00 (reference)
	Yes	0.99 (0.86, 1.14)	1.45 (1.21, 1.73)	1.07 (0.90, 1.27)	1.52 (1.22, 1.88)	0.81 (0.62, 1.05)	1.31 (0.96, 1.79)

Values are presented as adjusted odds ratio (95% confidence interval).

1 Multivariate logistic analysis included all variables in the model.
