# Supplementary material for: Trends in prediabetes and diabetes prevalence and associated risk factors in Vietnamese adults
Source: Epidemiol Health. 2020 May 11;42:e2020029. doi: 10.4178/epih.e2020029 (PMC7644943; doi:10.4178/epih.e2020029)
Supplement: Supplementary Material 1. [file epih-42-e2020029-suppl1.pdf]

Volume: 42, Article ID: e2020029
https://doi.org/10.4178/epih.e2020029

Supplementary Material 1. Age-standardized prevalence of diabetes among study participants[1] *(Based on FPG only)*

**Year**	**2011**	**2012**	**2013**	**2014**	**2015**	**2016**	**2017**	**APC %**	**P_{trend}**
No. respondents	1530	2998	3982	994	1251	981	989		
No. with diabetes	146	185	293	115	162	125	108		
Crude prevalence	9.5±0.8	6.2±0.4	7.4±0.4	11.6±1.0	12.9±0.9	12.7±1.1	10.9±1.0	11.60	0.1
Adj. prevalence	7.9±0.7	5.6±0.4	6.0±0.4	10.2±1.0	11.5±0.9	11.0±1.0	10.4±1.0	12.44	0.1
Age group									
45 - 49	6.0±1.4	4.2±0.7	4.5±0.6	6.8±1.6	7.1±1.4	7.7±1.6	6.6±1.4	10.74	0.1
50 - 54	6.7±1.4	4.7±0.9	3.9±0.8	8.2±2.0	10.8±2.0	8.1±2.2	10.9±2.2	16.36	<0.001
55 - 59	7.0±1.5	6.4±1.0	6.4±0.9	15.0±2.4	13.0±2.0	11.8±2.4	9.2±2.0	9.45	0.1
60 - 64	11.0±2.0	8.0±1.2	9.8±1.3	11.9±2.6	12.4±2.4	20.2±3.0	12.3±2.6	14.72	0.1
65 - 69	15.9±1.9	8.6±1.2	12.2±1.1	16.3±2.6	24.9±3	17.5±2.8	22.3±3.7	13.50	0.1
Sex									
Female	7.7±0.8	5.3±0.5	5.7±0.4	9.9±1.1	10.5±1.0	9.4±1.1	9.7±1.1	12.13	0.1
Male	8.6±1.6	6.8±1.0	7.0±0.8	11.5±2.1	14.6±2.0	15.5±2.4	12.9±2.2	12.12	0.1
Educational levels									
Illiteracy to secondary school (grade 9 and below)	8.3±1.0	5.9±0.5	7.5±0.6	10.3±1.4	11.8±1.2	12.1±1.4	10.9±1.3	12.25	<0.001
High school (grade 10-12)	6.1±1.2	4.8±0.8	3.8±0.6	9.8±1.6	12.0±1.8	10.7±1.9	10.5±1.8	18.67	0.1
College or more	6.7±2.0	4.9±1.2	4.1±0.9	10.5±2.3	10.2±2.5	6.2±2.3	8.1±2.8	11.22	0.3
Region									
Rural	N/A	4.8±0.7	6.1±0.8	N/A	N/A	10.5±1.4	11.7±1.4	N/A	N/A
Urban	7.9±0.7	6±0.5	6.0±0.4	10.2±1.0	11.5±0.9	11.8±1.5	9.2±1.3	11.72	0.3

[1] Data are percentage (±Standard Error). Direct age adjustment of the data was done for the Vietnamese population aged ≥45 years in the year 2009. APC: annual percent change. FPG: The fasting plasma glucose test
