# Supplementary material for: Trends in prediabetes and diabetes prevalence and associated risk factors in Vietnamese adults
Source: Epidemiol Health. 2020 May 11;42:e2020029. doi: 10.4178/epih.e2020029 (PMC7644943; doi:10.4178/epih.e2020029)
Supplement: Supplementary Material 2. [file epih-42-e2020029-suppl2.pdf]

Volume: 42, Article ID: e2020029
https://doi.org/10.4178/epih.e2020029

Supplementary Material 2. Age-standardized prevalence of prediabetes among study participants[1] *(Based on FPG only)*

Year (N=12725)	2011	2012	2013	2014	2015	2016	2017	APC (%)	P_{trend}
No. respondents	1530	2998	3982	994	1251	981	989		
No. with prediabetes	469	632	1248	540	659	489	522		
Crude prevalence overall	34.7±1.2	24.1±0.8	34.4±0.8	56.3±1.6	54.8±1.4	53.0±1.6	53.1±1.6	13.04	<0.001
Adj. prevalence overall	32.6±1.3	23.0±0.8	33.1±0.8	55.2±1.7	55.2±1.5	51.8±1.7	53.2±1.6	14.35	<0.001
Age group									
45 - 49	26.5±2.6	20.5±1.4	30.6±1.4	50.4±3.3	52.8±2.8	48.0±3.0	47.2±2.9	12.88	<0.001
50 - 54	33.3±2.6	21.2±1.7	33.2±1.9	56.0±3.7	58.2±3.1	50.6±4.0	57.5±3.6	18.68	<0.001
55 - 59	34.9±2.8	22.6±1.7	32.4±1.7	59.1±3.3	57.0±3.0	55.1±3.7	58.3±3.4	16.06	<0.001
60 - 64	38.6±3.1	29.4±2.1	34.8±2.0	56.3±3.9	58.2±3.5	55.7±3.7	54.8±4.0	15.25	<0.001
65 - 69	39.5±2.5	29.7±1.9	40.8±1.6	60.7±3.5	47.8±3.5	57.7±3.6	50.0±4.4	15.20	<0.001
Sex									
Female	34.2±1.5	25.0±1.0	35.5±1.0	57.6±2.0	56.3±1.7	53.1±2.0	52.8±1.9	11.74	<0.001
Male	28.4±2.4	17.2±1.4	27.4±1.4	50.0±3.3	52.3±3.0	48.7±3.5	54.8±3.3	9.47	0.1
Educational levels									
Illiteracy to secondary school(grade 9 and below)	34.6±1.9	23.3±1.0	33.8±1.1	55.6±2.5	57.3±1.9	52.4±2.2	55.7±2.2	15.07	<0.001
High school (grade 10-12)	30.6±2.2	22.4±1.6	33.4±1.5	54.9±2.9	51.9±2.8	50.6±3.3	49.5±2.9	13.13	<0.001
College or more	29.3±3.4	23.9±2.3	29.9±2.1	57.8±3.8	52.3±4.3	51.9±4.7	49.2±4.7	14.98	0.1
Region									
Rural	N/A	20.8±1.3	37.4±1.7	N/A	N/A	51.5±2.4	57.7±2.3	N/A	N/A
Urban	32.6±1.3	24.2±1.0	31.7±0.9	55.2±1.7	55.2±1.5	52.3±2.5	48.5±2.3	14.67	<0.001

[1] Data are percentage (±Standard Error). Direct age adjustment of the data was done for the Vietnamese population aged ≥45 years in the year 2009. APC: annual percent change. FPG: The fasting plasma glucose test
