# Supplementary material for: Trends in prediabetes and diabetes prevalence and associated risk factors in Vietnamese adults
Source: Epidemiol Health. 2020 May 11;42:e2020029. doi: 10.4178/epih.e2020029 (PMC7644943; doi:10.4178/epih.e2020029)
Supplement: Supplementary Material 3. [file epih-42-e2020029-suppl3.pdf]

Volume: 42, Article ID: e2020029
https://doi.org/10.4178/epih.e2020029

Supplementary Material 3. Binary logistic regression analysis: related factors of prediabetes and diabetes, compared to adults with normal glucose tolerance, 2011-2017[1].

	**Both**		**Women**		**Men**	
	aOR	95%CI	aOR	95%CI	aOR	95%CI
Age group						
45 - 49	1.00		1.00		1.00	
50 - 54	1.08	0.94-1.24	1.10	0.93-1.30	1.03	0.80-1.32
55 - 59	1.13	0.99-1.29	1.17	1.00-1.38	1.06	0.84-1.34
60 - 64	1.37	1.19-1.58	1.60	1.35-1.90	1.01	0.79-1.29
65 - 69	1.49	1.30-1.70	1.61	1.37-1.89	1.26	1.00-1.58
Gender						
Women	1.00		-	-	-	-
Men	0.82	0.74-0.91	-	-	-	-
Educational levels						
Illiteracy to secondary school(grade 9 and below)	1.00		1.00		1.00	
High school (grade 10-12)	0.97	0.88-1.08	1.04	0.91-1.18	0.88	0.74-1.04
College or more	1.01	0.87-1.16	1.02	0.85-1.24	0.99	0.79-1.22
Region						
Rural	1.00		1.00		1.00	
Urban	1.09	0.99-1.21	1.11	0.98-1.25	1.07	0.90-1.28
Obesity (BMI≥25)						
No	1.00		1.00		1.00	
Yes	1.48	1.33-1.65	1.40	1.23-1.59	1.74	1.42-2.14
Waist-hip ratio						
Normal	1.00		1.00		1.00	
Large	1.23	1.08-1.39	1.09	0.88-1.34	1.36	1.15-1.60
Hypertension						
No	1.00		1.00		1.00	
Yes	1.17	1.10-1.24	1.16	1.08-1.25	1.16	1.05-1.29
Abdominal Obese						
No	1.00		1.00		1.00	
Yes	1.17	1.06-1.30	1.26	1.12-1.42	0.93	0.76-1.15
Dyslipidemia						
No	1.00		1.00		1.00	
Yes	1.11	0.98-1.26	1.18	1.01-1.37	0.96	0.76-1.20

[1] Diabetes and prediabetes was combined for the outcome. aOR, adjusted odds ratio. Multivariate logistic analysis included all variables in the model.
